# Whale breaching says it loud and clear

**DOI:** 10.7554/eLife.55722

**Published:** 2020-03-11

**Authors:** Alexander J Werth, Charles L Lemon

**Affiliations:** Department of Biology, Hampden-Sydney CollegeHampden-SydneyUnited States

**Keywords:** locomotion, reproduction, communication, breaching, cetaceans, animal behavior, Other

## Abstract

A whale leaping above the surface expends an enormous amount of energy, displaying its health and strength to peers and potential mates.

**Related research article** Segre PS, Potvin J, Cade DE, Calambokidis J, Di Clemente J, Fish FE, Friedlaender AS, Gough WT, Kahane-Rapport SR, Oliveira C, Parks SE, Penry GS, Simon M, Stimpert AK, Wiley DN, Bierlich KC, Madsen PT, Goldbogen JA. 2020. Energetic and physical limitations on the breaching performance of large whales. *eLife* **9**:e51760. doi: 10.7554/eLife.51760

From marine biologists on their first field trip to the most jaded sailors, witnessing whales leaping above the sea surface – a behavior called breaching – rarely fails to elicit child-like wonder (Würsig and Whitehead, 2018). But why do whales breach? Is it, for instance, to dislodge external parasites, or could it simply be for play? Since these cetaceans often jump near each other, scientists have long thought that breaching might be a form of communication, a way for a whale to say: “Look how strong I am, I can propel my huge body right out of the water!” Beyond physical strength, this would also convey information about the animal’s genetic fitness – in other words: “You really should consider me as a potential mate!”

Gazelles that conspicuously leap directly in front of predators, peacocks which sport ridiculously huge tails, and even humans who smoke cigarettes or cover their bodies with tattoos are all prime examples of so-called ‘handicap signals’. These traits or behaviors are energy consuming or risky, but they demonstrate that the individual can bear these costs and still thrive (Zahavi, 1977). For a signal to be ‘honest’, however, it must entail a genuine expense to the animal. Until recently, it was not clear how much energy a breach would truly require from a whale, and how this was limited by the length of the animal, its swimming speed and other factors (Figure 1).

**Figure 1.**
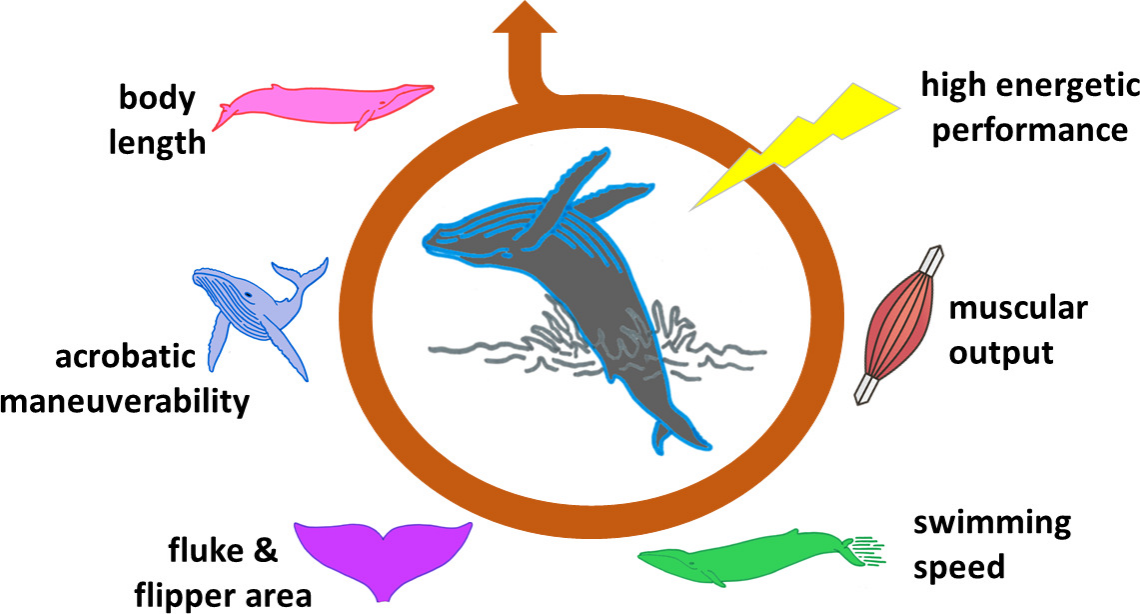
The ability of a whale to breach is influenced by many factors. Breaching is an energetically expensive behavior which depends on a number of interconnected elements. The length of the animal is one of the most limiting factors, affecting, for instance, body maneuverability. Shorter whales require lower swimming speeds to breach, and therefore lower muscle power. The surface area of flukes and flippers also influences how fast a whale can swim and turn. These and other features determine whether the animal has the capacity to expend a large amount of energy in a very short time (high energetic performance), and is therefore able to breach.

Now, in eLife, Paolo Segre from Stanford University and colleagues based in Italy, Portugal, the United States and Denmark report the results of a study on the energetic limits of breaching (Segre et al., 2020). The 18-member team used long poles and suction cups to temporarily attach sensors onto the bodies of five species of whales. The instruments recorded 187 breaches, tracking water depth and temperature as well as capturing the position of the whales over time and video clips before and during the jumps (Goldbogen et al., 2017; Cade et al., 2018).

Using these data to reconstruct the trajectory of breaching whales showed, for example, that young humpback whales often roll on their sides as they exit the water, while the adults usually emerge right-side up or upside-down. Swimming speeds reached a brisk 8–9 meters per second, but the large amounts of energy required for each breach did not stop some of the whales from leaping repeatedly: indeed, one juvenile humpback breached 52 times in just over four hours.

To calculate how much energy a whale spends on a breach, Segre et al. considered a series of factors that included metabolic rates, the energy needed for muscular contractions (Fish, 1998), the frequency of the tail stroke, and variables that influence water resistance (such as viscosity). Calculations showed that the energy costs of breaching depended on muscle power, which in turn is related to the length of the whale (Kahane-Rapport and Goldbogen, 2018; Gough et al., 2019). Large specimens must swim faster to clear the surface, requiring their muscles to generate tremendous power: a 15-meter-long humpback whale needs nearly ten times more energy per breach than a whale that is 8 meters long. In fact, in the few seconds that it takes to breach, the 15-meter whale can expend as much energy as a 60-kilogram human would during a marathon. Segre et al. therefore contend that a breach may represent the “most expensive burst maneuver” in all of nature, pushing the boundaries of muscular performance and providing an honest signal of a whale’s general health.

Overall, it is the length of a whale (rather than its mass) that appears to be the biggest obstacle to breaching: this may explain differences in breaching behavior between species of similar mass, but distinct builds. Blue whales, the longest species, are limited by how much power their muscles can deliver in short bursts and by the hydrodynamics of their tails. On the other hand, right whales, another large species, have short and chunky bodies, wider tails, and a thick, buoyant fat layer, all of which could help with breaching. In the end, species like humpbacks – famous for their agility as much as their complex social structure (Segre et al., 2019) – might be best suited to breaching. Regardless, there is no doubt that breaching comes at great expense for all types of whales: so next time you marvel at a leaping cetacean, consider the breach’s dazzling metabolic cost as well as its eye-widening beauty.
